# Melatonin modulates IL-1β-induced extracellular matrix remodeling in human nucleus pulposus cells and attenuates rat intervertebral disc degeneration and inflammation

**DOI:** 10.18632/aging.102472

**Published:** 2019-11-26

**Authors:** Yan Zhang, Fan He, Zhi Chen, Qihang Su, Meijun Yan, Qiang Zhang, Jun Tan, Lie Qian, Yingchao Han

**Affiliations:** 1 Department of Spinal Surgery, Shanghai East Hospital, Tongji University, School of Medicine, Shanghai 200120, China; 2 Department of Spine Surgery, Renji Hospital, School of Medicine, Shanghai Jiao Tong University, Shanghai 200127, China

**Keywords:** intervertebral disc degeneration, nucleus pulposus cells, melatonin, inflammation

## Abstract

The inflammatory-associated factors interleukin-1β (IL-1β), interleukin-6 (IL-6) and tumor necrosis factor-α (TNF-α) are widely reported to be associated with intervertebral disc (IVD) degeneration (IVDD). N-acetyl-5-methoxytryptamine (melatonin) is a natural hormone secreted by the pineal gland which has been shown to participate in several physiological and pathological progresses, such as aging, anti-inflammation, anti-apoptosis and autophagy regulation. However, the effects of melatonin on IVD remain unclear. In the present study, we treated human nucleus pulposus cells (NPCs) with melatonin and discovered that melatonin could modulate extracellular matrix (ECM) remodeling induced by IL-1β by enhancing collagen II and aggrecan expression levels and by downregulating matrix metalloproteinase-3 (MMP-3) levels. These findings were verified by western blot and immunofluorescence assays. Intraperitoneal injection of melatonin mitigated IVDD in the rat tail puncture model. X-ray and magnetic resonance imaging (MRI), as well as hematoxylin-eosin (H&E), Safranine O-Green, Alcian blue and Celium red staining methods were adopted to evaluate IVDD grades, the structural integrity of nucleus pulposus (NP) and annulus fibrosus (AF) and the damage and calcification of the cartilage endplate. Melatonin reduced inflammatory cell aggregation and the release of the inflammatory factors IL-1β, IL-6, TNF-α as determined by immunohistochemistry. In conclusion, the present study demonstrated that melatonin could modulate ECM remodeling by IL-1β in vitro and attenuate the IVDD and induction of inflammation in a rat tail puncture model in vivo. The data demonstrated that melatonin may contribute to the restoration processs of IVD following damage and may be used as a potential novel therapy for IVDD.

## INTRODUCTION

Intervertebral disc (IVD) degeneration (IVDD) is a disease that develops over age worldwide. The IVD begins to degenerate slowly following maturity of adolescence. The prevalence rate of IVDD-associated diseases is increasing every year. IVDD is directly associated with several factors, such as aging, genetic susceptibility, body weight, heavy load work and smoking [1, 2]. IVD is composed of the three following main structures: nucleus pulposus (NP), annulus fibrosus (AF) and cartilage endplate (EP). NP is a flexible sphere located in the center of IVD and is surrounded by AF in the anterior, posterior, left and right directions. EP is attached on the superior and inferior NP [3]. The factors that damage NP, AF or EP may lead to the degeneration of IVD. NP plays the core role in the function of IVD, which is composed of nucleus pulposus cells (NPCs) and extracellular matrix (ECM) components. NP is avascular tissue and gains nutrients by endplate diffusion. Oxidative stress, inflammation, cell starvation and mechanical force can lead to the dysfunction of NPCs and disrupt the balance of ECM synthesis and decomposition [4]. The regulation of the function of NPCs and the restoration of the balance of ECM metabolism is required for the delay of the IVDD progression.

N-acetyl-5-methoxytryptamine (melatonin) is a natural endocrine hormone mainly synthesized by the pineal gland in the brain. It is a highly pleiotropic regulator molecule that participates in several types of physiological function. The control of the circadian rhythm is the most important effect of melatonin, which is related with the light stimulus. The disorder of melatonin secretion can lead to physiological disorders in animals and plants [5]. In addition, melatonin further plays important roles in several diseases by scavenging free radicals, suppressing inflammatory factors, inhibiting oxidative stress, aging and by activating gene damage repair pathways [6–8]. Melatonin contributes to the prevention of memory impairment in aging by attenuating the alteration in the inflammation-associated protein levels [9]. Melatonin decreases the expression levels of inflammation and apoptotic markers in the lung of a senescence-accelerated mouse model [10]. Moreover, melatonin ameliorates rheumatoid arthritis by inhibiting TNF-α and IL-1β production, which is promoted via the downregulation of the PI3K/AKT, ERK and NF-κB signaling pathways, as well as the elevated expression of miR-3150a-3p [11]. Therefore, melatonin is functional in different types of disease by reducing the reaction of inflammatory factors as an adjuvant treatment. Inflammation is a widely accepted factor closely related to the initiation and development of IVDD and the potential application of melatonin as an anti-inflammatory treatment has been extensively investigated [11, 12]. It has already been shown that melatonin can protect NP cells against apoptosis, while it can promote mitophagy and ameliorate disc degeneration [13, 14]. However, whether melatonin can decrease the progression of IVDD by affecting the severity of inflammation remains unclear.

## RESULTS

### The cytotoxicity of melatonin or IL-1β on NPCs is considerably low

**Figure 1:**
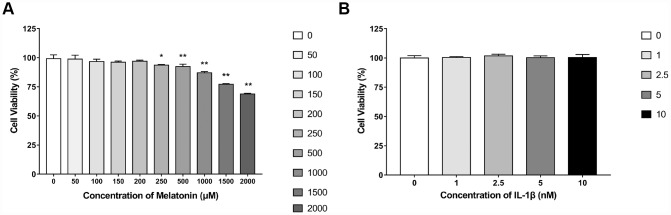
**Effects of gradient concentrations of melatonin or IL-1β on the viability of NPCs for 24h detected by CCK-8 assay.** (**A**) 0, 50, 100, 150, 200, 250, 500, 1000, 1500, 2000 μM melatonin treated NPCs for 24h (n=4). (**B**) 0, 1, 2.5, 5, 10 nM IL-1β treated NPCs for 24h (n=4). The values are expressed as mean ± SD. *P<0.05 vs control group; **P<0.001 vs control group.

NPCs were treated with 0, 50, 100, 150, 200, 250, 500, 1,000, 1,500 and 2,000 μM melatonin for 24 h and the viability of the cells was determined by the CCK-8 assay. The results revealed that when the concentration of melatonin was between 0 and 200 μM, no apparent influence was noted on NPC viability. The viability of the cells was only decreased by approximately 30% when the concentration was 2,000 μM, indicating that the cytotoxicity of melatonin on NPCs was low (Figure 1A). Therefore, 200 μM melatonin was used for 24 h as the appropriate processing condition. Since 0, 1, 2.5, 5 and 10 nM IL-1β exhibited no significant reduction on cell viability (Figure 1B), 5 nM IL-1β was selected to incubate NPCs for 24 h in the following experiments.

### Melatonin regulates extracellular matrix (ECM) synthesis and inhibits ECM remodeling initiated by IL-1β

**Figure 2:**
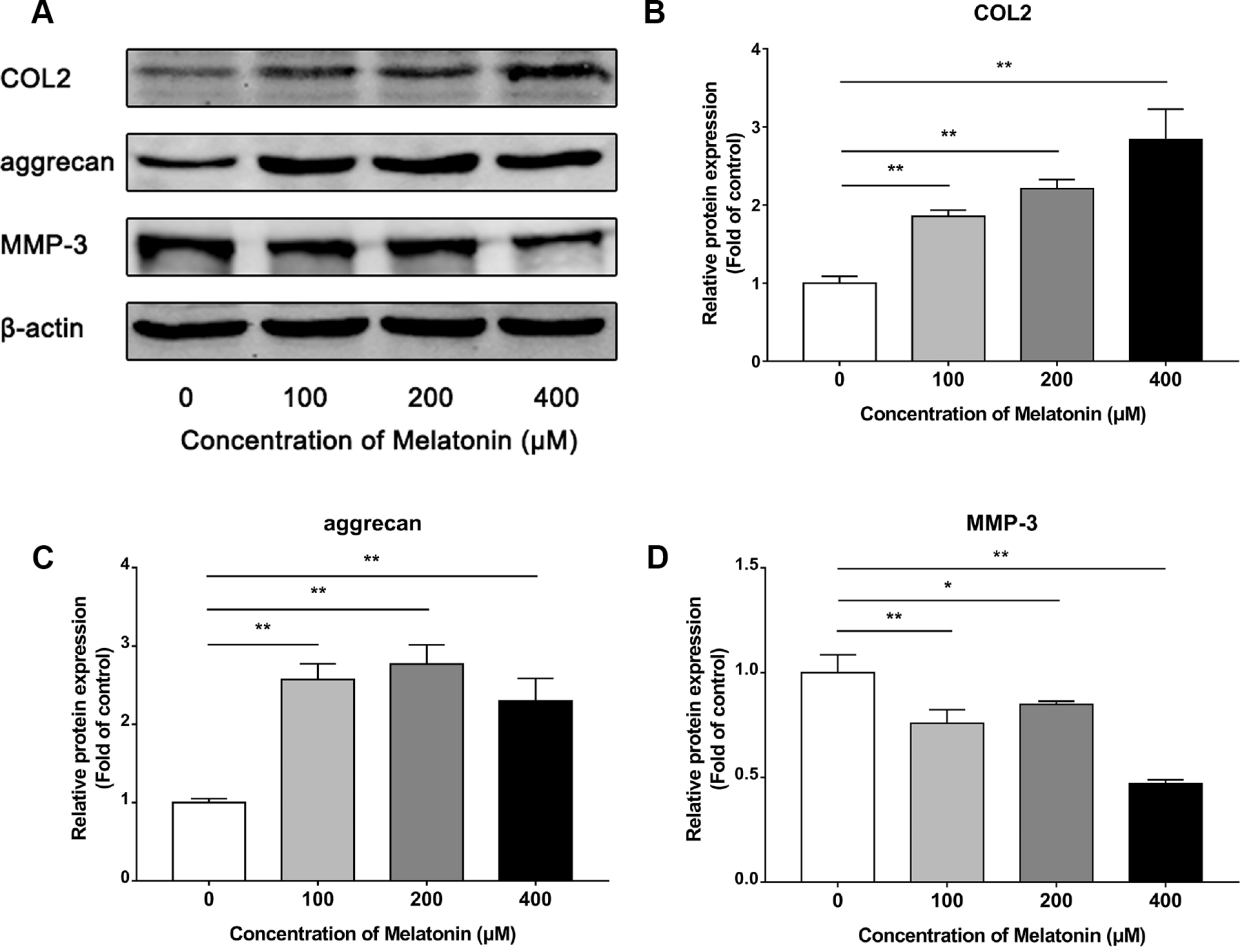
**Effects of 0, 100, 200, 400 μM melatonin for 24 h on collagen II (COL2), aggrecan and MMP-3 in NPCs.** (**A**) Protein levels of COL2, aggrecan, MMP-3. (**B**) Semi-quantitative analysis of COL2 levels (n=3). (**C**) Semi-quantitative analysis of aggrecan levels (n=3). (**D**) Semi-quantitative analysis of MMP-3 levels (n=3). The values are expressed as mean ± SD. *P<0.05 vs control group; **P<0.01 vs control group.

**Figure 3:**
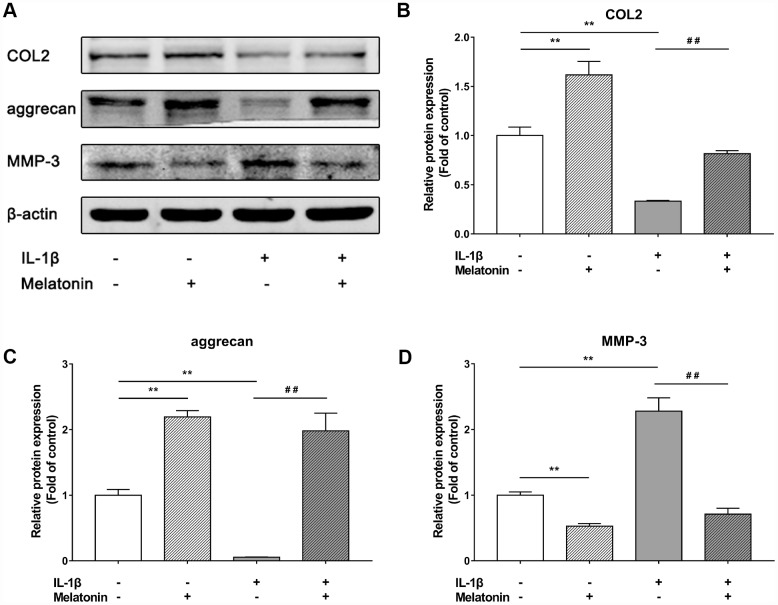
**Effects of 200 μM melatonin and 5 nM IL-1β for 24 h on collagen II (COL2), aggrecan and MMP-3 in NPCs.** (**A**) Protein levels of COL2, aggrecan, MMP-3. (**B**) Semi-quantitative analysis of COL2 levels (n=3). (**C**) Semi-quantitative analysis of aggrecan levels (n=3). (**D**) Semi-quantitative analysis of MMP-3 levels (n=3). The values are expressed as mean ± SD. **P<0.01 vs control group; ##P<0.01 vs IL-1β group.

**Figure 4:**
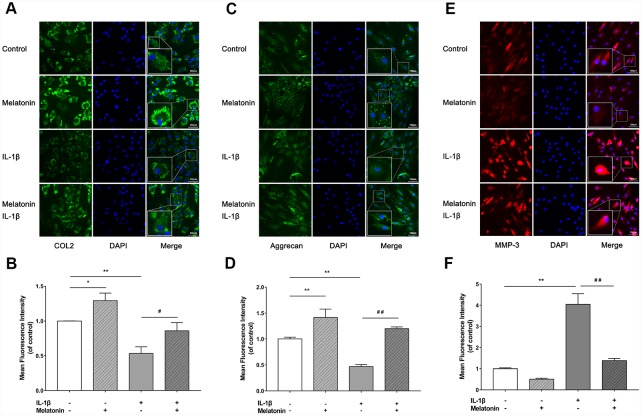
**Effects of 200 μM melatonin and 5 nM IL-1β for 24 h on collagen II (COL2), aggrecan and MMP-3 in NPCs detected by immunofluorescence.** (**A**) Representative images of immunofluorescence of COL2 in NPCs photographed by fluorescence microscopy (scale bar=100μm). (**B**) Semi-quantitative analysis of COL2 fluorescence intensity (n=3). (**C**) Representative images of immunofluorescence of aggrecan in NPCs photographed by fluorescence microscopy (scale bar=100μm). (**D**) Semi-quantitative analysis of aggrecan fluorescence intensity (n=3). (**E**) Representative images of immunofluorescence of MMP-3 in NPCs photographed by fluorescence microscopy (scale bar=100μm). (**F**) Semi-quantitative analysis of MMP-3 fluorescence intensity (n=3). The values are expressed as mean ± SD. *P<0.05 vs control group; **P<0.01 vs control group; ##P<0.01 vs IL-1β group.

Initially, NPCs were incubated with gradient concentration of melatonin and the levels of the ECM-associated proteins COL2, aggrecan and MMP-3 were examined. COL2 and aggrecan are two main components of the ECM that are responsible for retaining the fluids within the tissues and preserving the resilience and volume of NP. The abnormal reduction in the synthesis or the degradation of COL2 and aggrecan may lead to the failure of the NP structure and the development of IVDD. Matrix metalloproteinase (MMP) enzymes and a disintegrin and metalloproteinase with thrombospondin motifs (ADAMTS) are the main components responsible for the degradation of ECM, which is mediated by MMP-3, 9, 13 and ADAMTS-4, 5. Following treatment with melatonin, NPCs expressed additional levels of COL2 and aggrecan, while the levels of MMP-3 were decreased (Figure 2A–2D). Li et al demonstrated similar results indicating that melatonin could inhibit NPC extracellular matrix (ECM) remodeling via the melatonin membrane receptor-mediated activation of the PI3K-Akt pathway [15]. The results suggested that melatonin could protect NP by regulating the components of ECM. Furthermore, we used IL-1β to initiate the remodeling of ECM by inhibiting the synthesis of COL2, aggrecan and the activation of MMP-3, as demonstrated by WB and immunofluorescence assays (Figures 3–4). Following co-culture with both IL-1β and melatonin, NPCs exhibited significantly different expression of the aforementioned markers compared with the NPCs treated with IL-1β. The downregulated COL2 and aggrecan levels were restored by melatonin treatment and the upregulation of MMP-3 expression was weakened concomitantly, suggesting that melatonin could inhibit ECM remodeling caused by IL-1β. In conclusion, the data indicated that melatonin may protect IVD-induced inflammation from degeneration by regulating the ECM components of NP.

**Figure 5:**
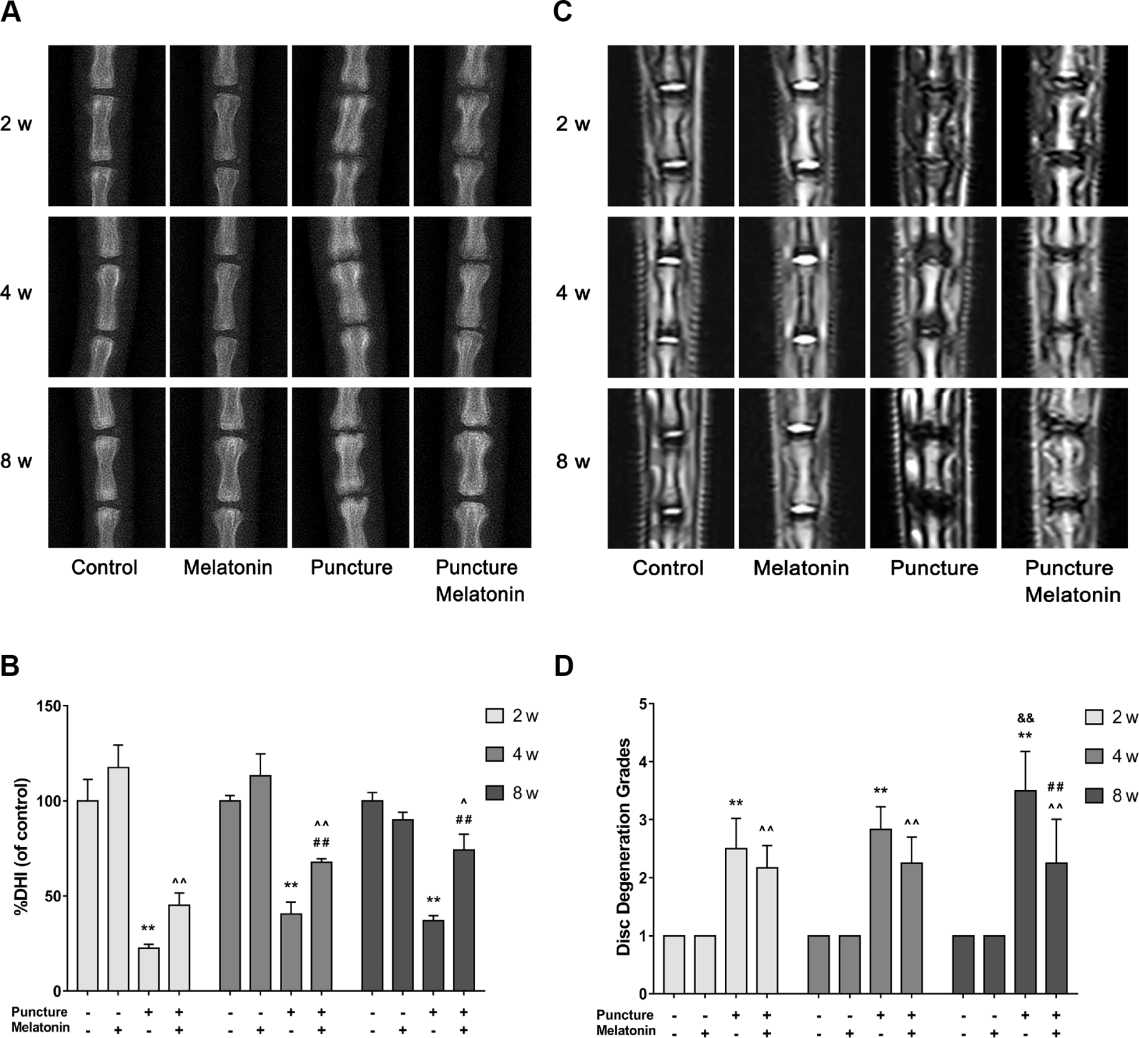
**Imaging characteristics of each group after 2, 4, 8 w according to the X-ray and MRI.** (**A**) Representative images of X-ray film. (**B**) Analysis of %DHI (DHI fold of control) based on X-ray (n=8). (**C**) Representative images of MRI. (**D**) Analysis of disc degeneration grades according to the Pfirrmann classification based on MRI (n=8). The values are expressed as mean ± SD. **P<0.01 vs control group; ^P<0.05 vs melatonin group; ^^P<0.01 vs melatonin group; ##P<0.01 vs puncture group; &&P<0.01 vs 2w puncture group.

Melatonin reduces the degree of IVDD in the rat tail puncture model. A total of 4 groups were prepared in our in vivo-study as mentioned above. These were the following: the control group, the melatonin group, the puncture group and the melatonin + puncture group. The rat tails were examined at the 2, 4 and 8 week periods by X-ray and MRI imaging in order to assess %DHI and disc degeneration grades based on the Pfirrmann classification. A total of 4 puncture points were used rather that only one point for each IVD level in order to ensure the modeling effect and avoid modeling failure caused by self-healing. The IVD that was punctured at the four avascular areas indicated apparent degeneration occurring at 2 weeks and swelling and inflammation of neighboring tissues. X-ray analysis indicated that IVDD was characterized by the height loss and the blur of the endplate boundary at the 2 week period. The height loss and endplate blur exhibited no amelioration at 8 weeks, while the structural integrity appeared to be damaged. This damage was identified as an insect bite-like defect, which could also be verified by subsequent histological staining (Figure 5A, 5B). Intraperitoneal injection of melatonin demonstrated that the IVD exhibited similar height loss and endplate blur at 2 weeks. However, at 8 weeks, the %DHI was doubled compared with that of the puncture group and was approximately equal to 70% of that of the control group. Moreover, no blur or structural failure of the endplate was noted at 8 weeks in the melatonin + puncture group (Figure 5A, 5B). The MRI images revealed that the signals of the T2-weighted imaging of NP and of the endplate in the puncture group were decreased compared with those noted in the normal structure at 2 weeks. Its appearance was grey. Further aggravation occurred at 8 weeks and the NP and endplate exhibited a black color, while the Pfirrmann grades were increased. In contrast to these findings, the Pfirrmann grades exhibited no progression from the period of 2 weeks to 8 weeks in the melatonin + puncture group, whereas the signal of the endplate remained normal at all time periods (Figure 5C, 5D). The melatonin group indicated no significant differences by both X-ray and MRI imaging compared with the corresponding images of the control group at either 2, 4 or 8 weeks. Thus, melatonin could inhibit the IVDD progression in the rat tail puncture model.

### Melatonin protects the structural integrity of IVD and attenuates inflammation

**Figure 6:**
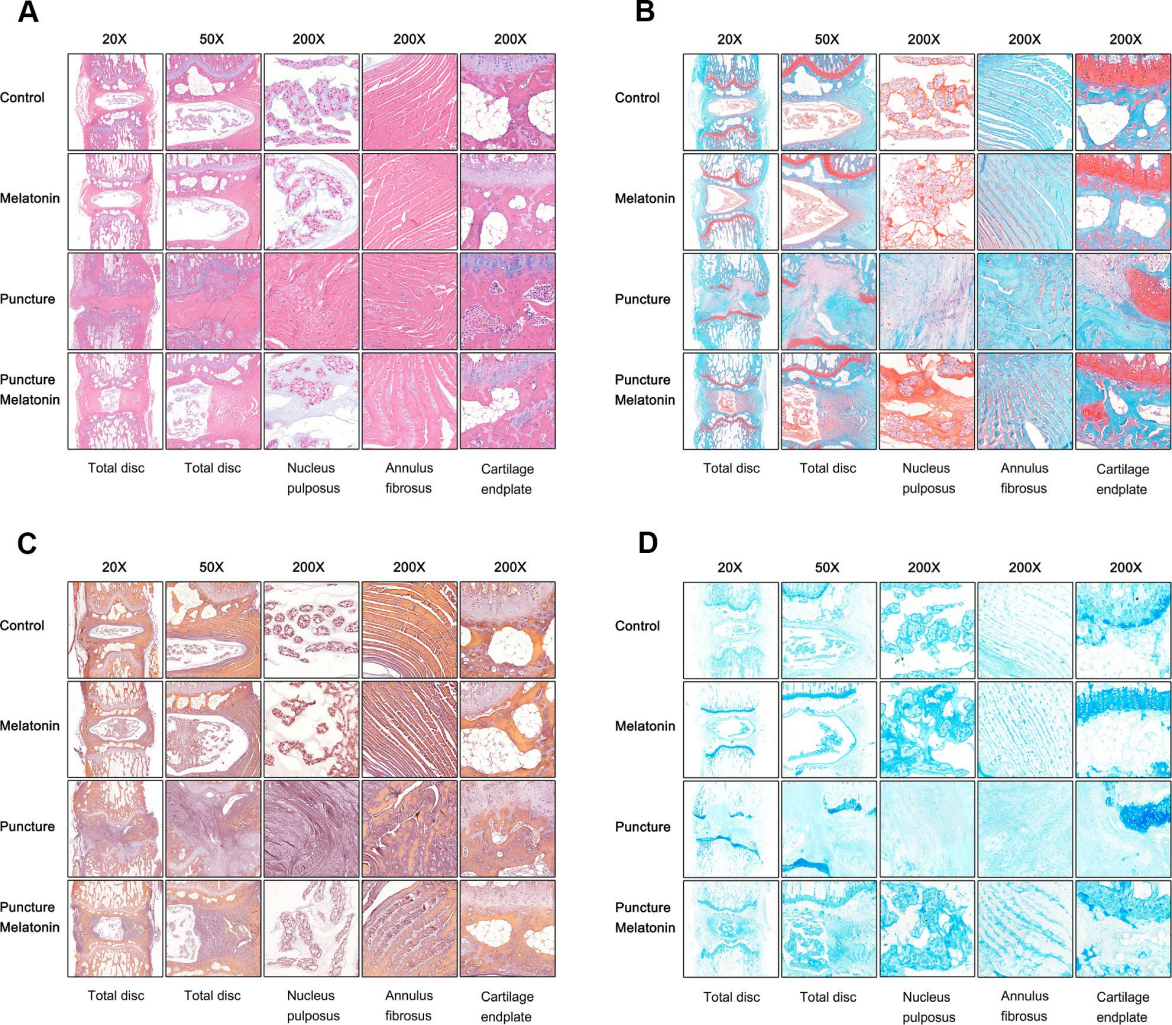
**Histological staining of each group 8w after puncture.** (**A**) Representative images of HE staining. (**B**) Representative images of Safranine O-Green staining. (**C**) Representative images of Celium red staining. (**D**) Representative images of Alcian blue staining.

**Figure 7:**
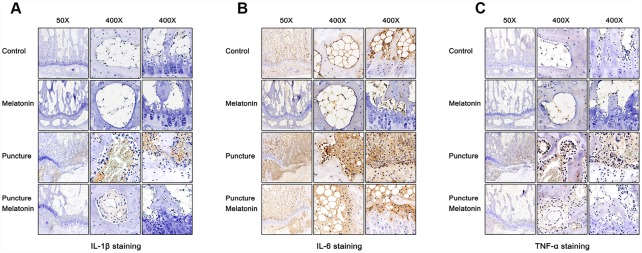
**Immunohistochemical staining of each group 8w after puncture.** (**A**) Representative images of IL-1β staining. (**B**) Representative images of IL-6 staining. (**C**) Representative images of TNF-α staining.

The rats were sacrificed and the discs with adjacent vertebral bodies were isolated, fixed, embedded and sliced. H&E, Safranine O-Green, Alcian blue and Celium red staining as well as immunochemistry were subsequently performed. It was noted that in the puncture group the structure of IVD indicated degenerated features. The NP vanished and was replaced by chaotic fibrous tissue, whereas the orderly arrangement of AF was destroyed and part of the endplate disappeared (Figure 6A–6D). Moreover, the niches within the endplate and the vertebral body were filled with inflammatory cells, expressing high levels of IL-1β, IL-6 and TNF-α (Figure 7A–7C), leading to the vitreous circulation of the local microenvironment of the intervertebral disc.

The melatonin + puncture group exhibited damaged vitreous circulation caused by melatonin. The inflammatory cells aggregated to the niches of the vertebral bodies and nearly disappeared. A limited number of these cells remained within the niches of the endplate and the expression levels of IL-1β, IL-6 and TNF-α were markedly decreased (Figure 7A–7C). Consequently, the integrity of IVD was retained. It was clearly shown that part of the NP was preserved, whereas the arrangement of AF resulted in hypertrophy and in a complete organization of the endplate structure (Figure 6A–6D). The melatonin group indicated no apparent changes in the histological staining, IL-1β, TNF-α expression levels and immunohistochemical examinations compared with the corresponding values of the control group. It is interesting to note that the expression levels of IL-6 in the endplate tissues were visibly decreased by melatonin in the melatonin group compared with those of the normal tissue. IL-6 expression levels were markedly increased in the endplate of the puncture compared with those of the control group, which could be suppressed by melatonin (Figure 7B). Therefore, melatonin protected the structural integrity of IVD and notably the integrity of the endplate by inhibiting inflammatory cell aggregation and inflammatory factor release.

## DISCUSSION

Inflammation plays important roles in the initiation and exacerbation of IVDD. IL-1β is the most important member of the IL-1 family and is highly expressed in degenerative IVD tissues. This cytokine is also involved in multiple pathological progresses, including inflammatory responses, matrix destruction, cellular apoptosis and oxidative stress [16]. Similarly, TNF-α levels are highly expressed in degenerative IVD tissues. This cytokine is also involved in matrix destruction, inflammatory responses, apoptosis, autophagy and cell proliferation [17]. IL-6, IL-8 and IL-17 are further reported to exert significant associations with the risk of lumbar degenerative disease [18–20]. In the present study, IL-1β was used to initiate the degenerative changes of NPCs as demonstrated by previous studies [21]. IL-1β downregulated the expression levels of COL2 and aggrecan, while it also upregulated the expression levels of MMP-3. These metabolic changes could be suppressed by melatonin. The present study demonstrated the effectiveness of melatonin in protecting NPC normal function and in inhibiting ECM remodeling induced by IL-1β.

NP is an avascular tissue and the EP becomes the main source of nutrition and of cytokine release. Nutritional disorder caused by dysfunction of endplate is considered to be one of the most important mechanisms involved in IVDD development [22]. The permeability of EP decreases to 50-60% and the solute transport is markedly inhibited in the degenerate disc [23]. Calcification of endplate can block nutrient and oxygen transport, thus leading to IVDD progression [24, 25]. In the current in vivo-study, a rat tail IVD puncture was performed that was parallel to the endplate in order to avoid endplate disruption. In the melatonin + puncture group, no evidence of endplate injury was noted by several detection methods including X-ray and MRI imaging and histological staining. However, in the puncture group, the border of endplate appeared to be blur on the X-ray films and its signal was black on the MRI images. These findings were confirmed by histological staining, where the absence of endplate tissue was directly shown. In addition, a very high number of inflammatory cells expressed high levels of IL-1β, IL-6 and TNF-α in the niches of both endplate and vertebral body of the puncture group. Moreover, high levels of inflammatory factors were released on the NPCs leading to the rapid degeneration of the IVD. In contrast to the puncture group, a low number of inflammatory cells was found within the endplate in the melatonin + puncture group, indicating the potent anti-inflammatory effect of melatonin. In summary, melatonin could protect the structure integrity of the endplate and regulate the function of NPCs by suppressing the inflammatory cell aggregation and the release of the inflammatory factors IL-1β, IL-6, TNF-α. These events in turn delayed the process of IVDD.

The reduction of inflammation can be used in the treatment of IVDD, since it can delay this process and reduce the pain inflicted by IVDD. Intradiscal delivery of celecoxib-loaded microspheres has been shown to restore intervertebral disc integrity in a preclinical canine model [12]. Celastrol is reported to reduce IL-1β induced matrix catabolism, oxidative stress and inflammation in human NPCs, and attenuate rat IVDD [26]. Recently, biomaterial anti-inflammatory Chitosan/Poly-γ-glutamic acid nanoparticles were also very effective for IVDD treatment by controlling inflammation and ECM remodeling [27]. Although melatonin has already been tested and verified to be a potent anti-inflammatory drug for a variety of other diseases [6, 10, 11], the present study demonstrated for the first time the anti-inflammatory effects of melatonin in the progression of IVDD, suggesting the important function of melatonin in the physiological repair of IVD and its potential in the treatment of IVDD-associated diseases.

Melatonin is a hormone secreted according to the circadian rhythm and is affected by both natural and artificial light. It has been reported that the light and noise in an intensive care unit (ICU) may disturb the sleep quality and affect the production of melatonin following major abdominal surgery, leading to the increase in serum IL-1, IL-6 and c-reactive protein (CRP) levels compared with those noted in patients treated with usual care [28]. Similarly, melatonin can induce efficient nerve regeneration with lower IL-1β levels when used in a dark environment compared with its use in a light environment. This was noted in a sciatic nerve injury rat model [29]. Briefly, the normal rhythmic secretion of melatonin can guarantee sufficient levels of serum concentration required for an anti-inflammatory effect. It was also found that night work can disturb the secretion of melatonin in healthy male workers, resulting in the lift of TNF, IL-1 and IL-6 in their saliva [30]. In the present study, melatonin could promote COL2 and aggrecan synthesis and inhibit MMP-3 production. This may explain the fact that sleep deprivation can weaken the restoration of IVD height and aggravate chronic low back pain [31–33]. Unhealthy sleep habits may obstruct the normal production of melatonin, resulting in the high levels of inflammatory factors in the body fluids. The chronic inflammatory condition may initiate the progression of several chronic diseases associated with inflammation, such as IVDD.

In conclusion, the present study demonstrated that melatonin could regulate the balance of ECM synthesis and its degradation under normal physiological state or under inflammatory conditions caused by IL-1β treatment of human NPCs. We further demonstrated that melatonin protected IVD structure integrity and delayed the progression of IVDD by inhibiting inflammatory cell aggregation and IL-1β, IL-6 and TNF-α release in a rat tail puncture model. With limited cytotoxicity, melatonin exhibited the potential to become a novel therapeutic agent for IVDD treatment.

## MATERIALS AND METHODS

### Reagents and antibodies

Fetal bovine serum (FBS) and penicillin-streptomycin were purchased from Gibco (Grand Island, NY, USA). Phosphate buffered solution (PBS) and Dulbecco’s modified Eagle’s medium/F12 (DMEM/F12) were purchased from Hyclone (South Logan, UT, USA). N-acetyl-5-methoxytryptamine (melatonin) was obtained from Sangon Biotech (Shanghai, China). IL-1β was purchased from R&D systems (Minneapolis, MN, USA). The cell Counting Kit-8 (CCK-8) was purchased from Beyotime (Beyotime Biotechnology, Shanghai, China). The antibodies against collagen II, aggrecan and MMP-3 were purchased from Abcam (Cambridge, MA, USA). The antibodies for β-actin were purchased from Cell Signaling Technology (Danvers, MA, USA).

### Experimental design

All NPCs were pretreated overnight with fresh DMEM/F12 containing 2% FBS prior to the experiments. In the first part of the in vitro-study, NPCs were treated with 0, 100, 200 and 400 μM melatonin for 24 h and the protein levels of collagen II, aggrecan and MMP-3 were detected by western blotting. In the second part of the in vitro-study, NPCs were divided into 4 groups as follows: a control group, an IL-1β group, a melatonin group and an IL-1β + melatonin group. The cells were incubated with 200 μM melatonin for 24 h and with 5 nM IL-1β for another 24 h. Subsequently, western blot and immunofluorescence assays were performed to monitor the changes in collagen II, aggrecan and MMP-3 expression. The in vivo-study comprised 48 rats that were punctured at the 2 caudal vertebral discs. These were the third and fourth coccygeal discs identified by counting the vertebrae from the sacral region and then dividing them into 2 groups randomly as follows: a control group and a melatonin group. The fifth and sixth unpunctured coccygeal discs were denoted as the self-control groups. Melatonin was dissolved initially with absolute ethanol and subsequently diluted with deionized water. The rats in the control group were injected intraperitoneally with melatonin (50 mg/kg/d) at 7pm daily [34]. The rats in the control group were administered intraperitoneally with the same amount of ethanol and deionized water at the same time. Finally, 4 groups were selected as follows: a control group, a puncture group, a melatonin group and a puncture + melatonin group. At 2, 4, 8 weeks following puncture, 8 rats were sacrificed and tested. The IVDD grades were evaluated by X-ray and MRI imaging. The structural integrity of NP and AF was shown by H&E staining, while the damage and calcification of cartilage endplate was detected by Safranine O-Green, Alcian blue and Celium red staining. The inflammation status was tested by immunochemistry.

### NPC isolation and culture

Human lumbar nucleus pulposus (NP) tissues were obtained from patients who underwent discectomies with written informed consent. The experimental protocol was approved by the Ethics Committee of the East Hospital affiliated to Tongji University, Shanghai, China.

NP tissues were separated according to the morphological difference of the cells as demonstrated by microscopy, followed by 0.25% trypsin (Gibco, USA) digestion at 37°C for 2 h and by 0.1% type II collagenase (Sigma, USA) digestion at 37°C for 6-8 h. Following centrifugation, NPCs were collected and cultured in a complete DMEM/F12 medium supplement with 20% FBS and 1% penicillin-streptomycin under the following hypoxic conditions: 1% O_2_, 5% CO_2_ and 94% N_2_ at 37°C in a humidified incubator. The second passage-NPCs were used throughout the experiments to prevent their dedifferentiation.

### Cell viability assay

CCK-8 assays were performed to detect the viability of NPCs according to the manufacturer’s instructions. NPCs were grown in 96-well plates at a density of 1.0-1.5 × 10^4^ cells/well. The cells were treated with 0, 50, 100, 150, 200, 250, 500, 1,000, 1,500 and 2,000 μM melatonin for 24 h in order to determine the appropriate treatment concentration. In addition, NPCs were incubated with 0, 1, 2.5, 5 and 10 nM IL-1β for 24 h in order to detect whether this concentration could influence their viability. Subsequently, 10 μl CCK-8 solution was added to 90 μl culture media, transferred to each well and incubated for 2-4 h at 37°C. At last, an auto-microplate reader (Multifunctional microplate reader SpectraMax M5, USA) was used to read the absorbance of each sample.

### Western blot analysis

All protein levels were determined by western blot analysis. The total protein of NPCs was extracted using the total protein extraction kit (Beyotime Biotechnology, Shanghai, China). The determination of the protein concentration was conducted using the BCA protein assay kit (Beyotime Biotechnology, Shanghai, China). The protein samples were mixed with loading buffer and boiled for 5-10 min. Subsequently, the proteins were separated using sodium dodecyl sulfate polyacrylamide (SDS) gels (8-15%) by polyacrylamide gel electrophoresis (PAGE). The proteins were transferred to 22 μm polyvinylidene fluoride membranes (PVDF membranes, Millipore, USA) using a wet-blotting method. Following blocking with 5% non-fat milk in Tris-buffered saline (TBS) containing 0.1% Tween-20 (TBST) for 1-2 h, the membranes were incubated overnight at 4°C with different primary antibodies (collagen II 1:1,000, aggrecan 1:500, MMP-3 1:1,500, β-actin 1:5,000) in 5% non-fat milk diluted in TBST. Following washing for 3 times in TBST, the membranes were incubated with the corresponding secondary antibodies (Cell Signaling Technology, USA) for 1 h at 37°C. The detection of the bands was performed using the Li-Cor Odyssey 9120 Infrared Imaging System (USA). The intensity of bands was quantified by the Image-Pro Plus 6.0 software (Media Cybernetics, USA).

### Immunofluorescence

NPCs were cultured in 24-well plates (4x10^4^cells/well), treated as mentioned above and fixed with fresh 4% paraformaldehyde for 15-20 min. Following washing with PBS that contained 0.1% Tween-20 (PBST), the cells were incubated with 0.2% Triton X-100 for 15 min. The samples were blocked with 5% goat serum for 30-60 min and the NPCs were treated with the primary antibody against collagen II (1:100), aggrecan (1:50) and MMP-3 (1:100) overnight at 4°C, followed by incubation with FITC-conjugated secondary antibodies for 1 h at 37°C. The fluorescent images were obtained using a fluorescence microscope (Leica DMI 3000B, Germany) and the intensity was quantified by the Image-Pro Plus 6.0 software.

### The annulus fibrosus needle puncture model

All surgical interventions, treatments and animal care procedures were performed in strict accordance with the Animal Care and Use Committee of the Tongji Medical University. A total of 48 female Sprague-Dawley rats were purchased from the Shanghai SLAC Laboratory Animal CO. LTD (Shanghai, China) and raised to a final weight range of 200-220 g. All rats were anesthetized with 4% chloral hydrate (10 ml/Kg, Sangon Biotech, Shanghai, China) by intraperitoneal injection. The third and fourth coccygeal discs were located by counting the vertebrae from the sacral region. The discs were punctured with a 26-gauge sterile needle in a parallel direction to the endplates from the four avascular zones through the skin and ligament towards the NP. The needle was rotated at 360° and held in that position for 30s, while the full layers of the AF were damaged. The rats were categorized into specific groups, treated and examined at specific time points as described above in the experimental design part.

### X-ray film and magnetic resonance imaging analysis

The X-ray film was obtained on an X-ray system (uDR 588i, United Imaging, Shanghai, China) and used to evaluate disc gross appearance and disc height status. The Disc Height Index (DHI) was adopted to assess disc height loss after modeling according to the method proposed by Masuda (Supplementary Figure 1) [35]. The formula used was the following: DHI=2(DH1+ DH2+DH3)/[(PV1+PV2+PV3)+(DV1+DV2+DV3)]

MRI imaging was performed on an MRI system (uMR 770, United Imaging, Shanghai, China) to evaluate disc degeneration grades according to the Pfirrmann classification (Supplementary Table 1), ranging from grade I (normal) to grade IV (advanced degeneration) [36].

### Histological staining

The isolated spines and adjacent vertebral bodies were fixed in 4% paraformaldehyde, decalcified, dehydrated, cleared with dimethylbenzene and embedded in paraffin (Tissue Tek processor and Leica embedder, Buffalo Grove, IL). The sections (5 μm) were stained with either Hematoxylin and Eosin, Safranin O, Alcian blue or Celium red (Fisher Scientific, Pittsburgh, PA) using standard procedures. The images were acquired under a 20× 50× and 200× magnification using the light microscopy mode of a fluorescence microscope (Leica DM 6000B, Germany).

### Immunohistochemical examination

Isolated spines and adjacent vertebral bodies were fixed in 4% paraformaldehyde, decalcified, dehydrated, cleared with dimethylbenzene and embedded in paraffin (Tissue Tek processor and Leica embedder, Buffalo Grove, IL). The sections (5 μm) were incubated with 3% hydrogen peroxide to block endogenous peroxidase activity for 10 min and 5% bovine serum albumin was used to block nonspecific binding sites for 30 min at 37°C. Following incubation with the primary antibody (IL-1β 1:1,000, IL-6 1:800, TNF-α 1:1,000, Servicebio, Wuhan, China) overnight at 4°C, the sections were treated with diaminobenzidine-based peroxidase substrate (DAB Horseradish Peroxidase Color Development Kit, Beyotime Biotechnology, Shanghai, China) to visualize the immunoreactivity. The images were acquired under 50× and 400× magnification using the light microscopy mode of the fluorescence microscope and the intensity was quantified by the Image-Pro Plus 6.0 software.

### Statistical analysis

All experiments were repeated at least three times. Statistical analyses were performed using the Statistical Product and Service Solutions 16.0 software (SPSS Inc., Chicago, USA). The data were expressed as mean ± standard deviation (SD). The statistical significance between groups was analyzed using one-way analysis of variance (ANOVA), and a P < 0.05 was considered for significant differences.

## Supplementary Material

Supplementary Figure 1

Supplementary Table 1
