# Supplementary material for: Melatonin modulates IL-1β-induced extracellular matrix remodeling in human nucleus pulposus cells and attenuates rat intervertebral disc degeneration and inflammation
Source: Aging (Albany NY). 2019 Nov 26;11(22):10499–512. doi: 10.18632/aging.102472 (PMC6914432; doi:10.18632/aging.102472)
Supplement: Supplementary Figure 1 [file aging-11-102472-s002..pdf]

SUPPLEMENTARY FIGURE

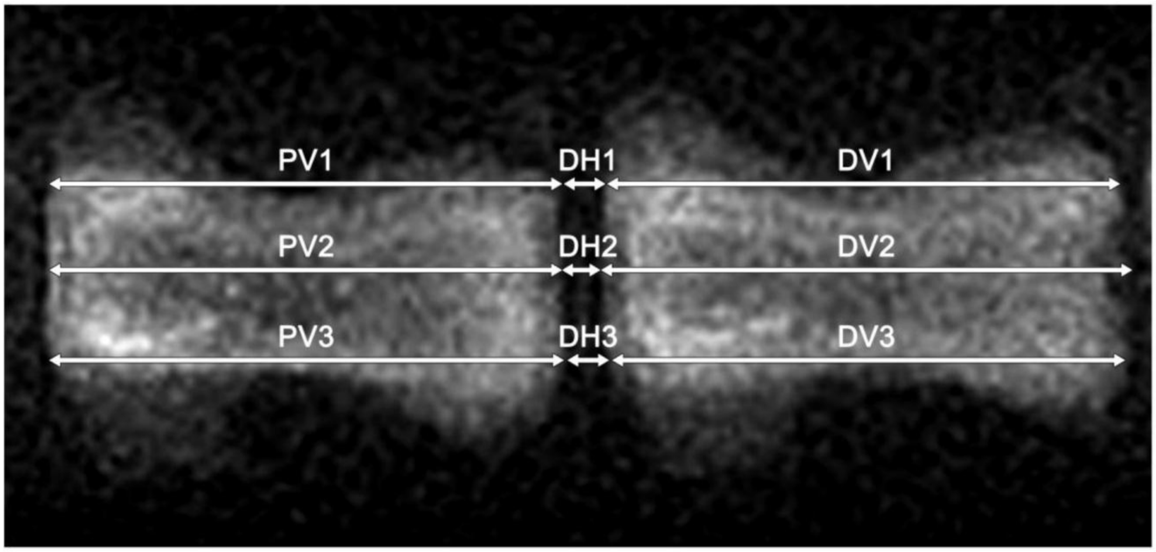

Supplementary Figure 1. Measurement of disc height on X-ray film (PV: proximal vertebral body; DH: disc height; DV: distal vertebral body).
