# Supplementary material for: Melatonin modulates IL-1β-induced extracellular matrix remodeling in human nucleus pulposus cells and attenuates rat intervertebral disc degeneration and inflammation
Source: Aging (Albany NY). 2019 Nov 26;11(22):10499–512. doi: 10.18632/aging.102472 (PMC6914432; doi:10.18632/aging.102472)
Supplement: Supplementary Table 1 [file aging-11-102472-s001..pdf]

SUPPLEMENTARY TABLE

Supplementary Table 1. Modified MRI grading according to Pfirrmann.

Grade	Structural changes within NP	Intervertebral disc height
I	Homogenous and bright	Normal
II	Heterogenous	Normal
III	Heterogenous and grey	Decreased
IV	Heterogenous and black	Decreased or collapsed
